# Supplementary material for: Rho GTPase activity crosstalk mediated by Arhgef11 and Arhgef12 coordinates cell protrusion-retraction cycles
Source: Nat Commun. 2023 Dec 15;14:8356. doi: 10.1038/s41467-023-43875-y (PMC10724141; doi:10.1038/s41467-023-43875-y)
Supplement: Supplementary file 1 — Supplementary Information [file 41467_2023_43875_MOESM1_ESM.pdf]

Supplementary Information for

Rho GTPase activity crosstalk mediated by Arhgef11 and Arhgef12 coordinates cell protrusion-retraction cycles

Suchet Nanda[1,2], Abram Calderon[1,2], Arya Sachan[1], Thanh-Thuy Duong[1,2], Johannes Koch[3], Xiaoyi Xin[4], Djamschid Solouk-Stahlberg[1,2], Yao-Wen Wu[4], Perihan Nalbant[3,*], Leif Dehmelt[1*]

[1]Fakultät für Chemie und Chemische Biologie, TU Dortmund University, 44227 Dortmund, Germany

[2]Department of Systemic Cell Biology, Max Planck Institute of Molecular Physiology, 44227 Dortmund, Germany

[3]Department of Molecular Cell Biology, Center of Medical Biotechnology, University of Duisburg-Essen, 45141 Essen, Germany

[4] SciLifeLab and Department of Chemistry, Umeå Centre for Microbial Research, Umeå University, 90187 Umeå, Sweden

*Corresponding authors: perihan.nalbant@uni-due.de, leif.dehmelt@tu-dortmund.de

Contents

Supplementary Figures 1-6

Supplementary Figures

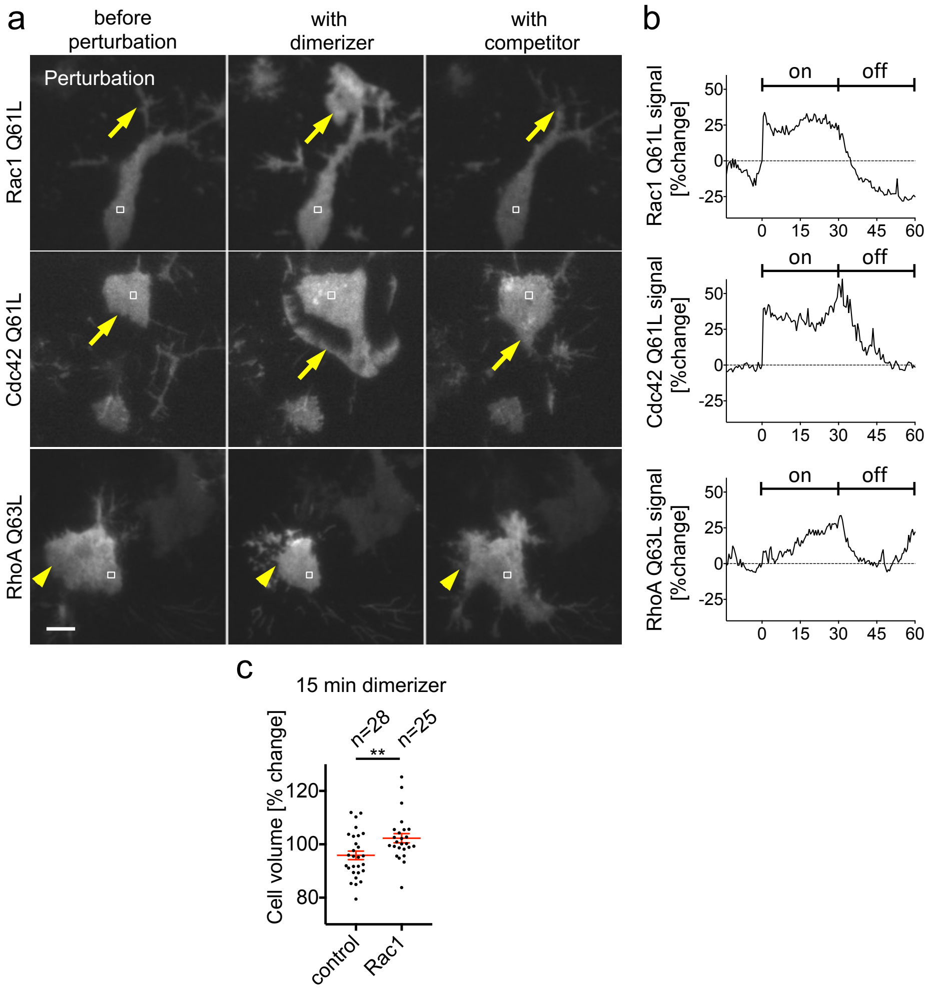

Supplementary Figure 1: Rapid perturbation of Rho GTPase activity in living cells via small molecule induced plasma membrane recruitment. A schematic for the small molecule dimerizer based strategy to introduce rapid Rho GTPase activity perturbations is shown in Fig. 1a. **a:** Representative frames from TIRF microscopy time series of dominant positive mutants of mTurquoise2-fused Rho GTPases, lacking the C-terminal plasma membrane targeting sequence. Images shown were obtained 30s before, 24 min during and 24min after application of the SLF'-TMP dimerizer to Neuro-2a neuroblastoma cells. Yellow arrows point to cell areas that reversibly generate protrusions during Rac1 or Cdc42 activation, and yellow arrowheads point to areas that undergo reversible retraction during RhoA activation. Corresponding images that show an actin-based reporter for the GTPase-induced changes in cytoskeletal dynamics are shown in Fig. 1b. b: Typical dynamics of chemical dimerizer-induced plasma membrane recruitment and competitor-induced reversal, corresponding to white boxes in a. **c**: Quantification of the change in cell volume induced by the 15 minutes application of the SLF'-TMP dimerizer, measured via scanning confocal microscopy of the volume marker mCherry. **: $P<0.01$; Student's t-Test. Error bars represent standard error of the mean. Scale bar: 10 µm; 0.26µm/pixel. All statistical tests were two-sided. Source data are provided as a Source Data file.

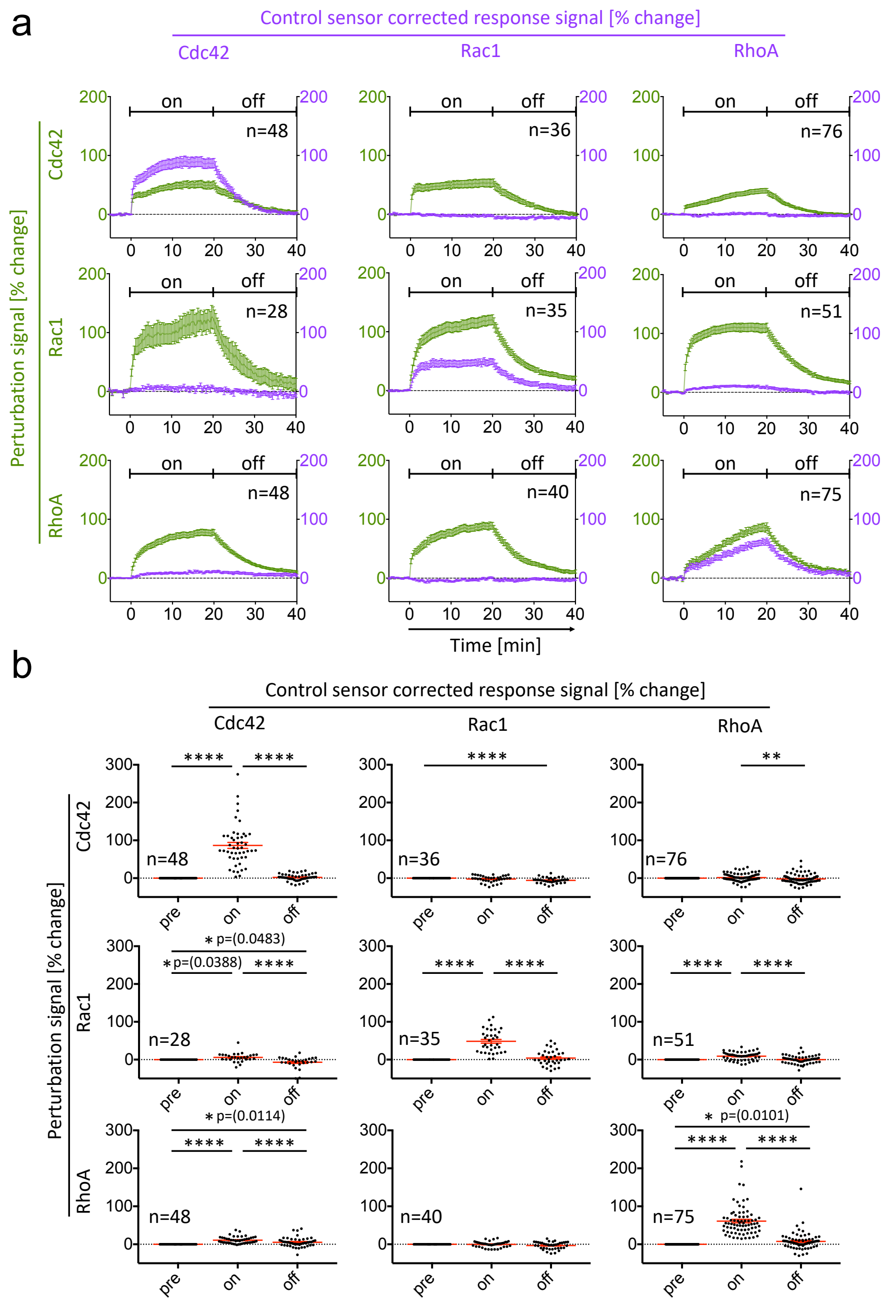

Supplementary Figure 2: Analysis of crosstalk between the the major Rho family GTPases Cdc42, Rac and Rho in living cells. a: Average perturbation and control-corrected activity sensor signal kinetics for all crosstalk combinations. **b:** Statistical analysis of average sensor signal changes during and after Rho GTPase activity perturbation. For these analyses, responses at time points before (pre), and 20 minutes after addition of dimerizer (on) or competitor (off) were considered. All observations and measurements are based on at least 3 independent repetitions with a total of at least 28 cells per condition (exact numbers of cells are indicated in individual panels). ****: $P<0.0001$; ***: $P<0.001$; **: $P<0.01$; *: $P<0.05$; One-way ANOVA, Tuckey's post test; Error bars represent standard error of the mean. All statistical tests were two-sided. Source data are provided as a Source Data file.

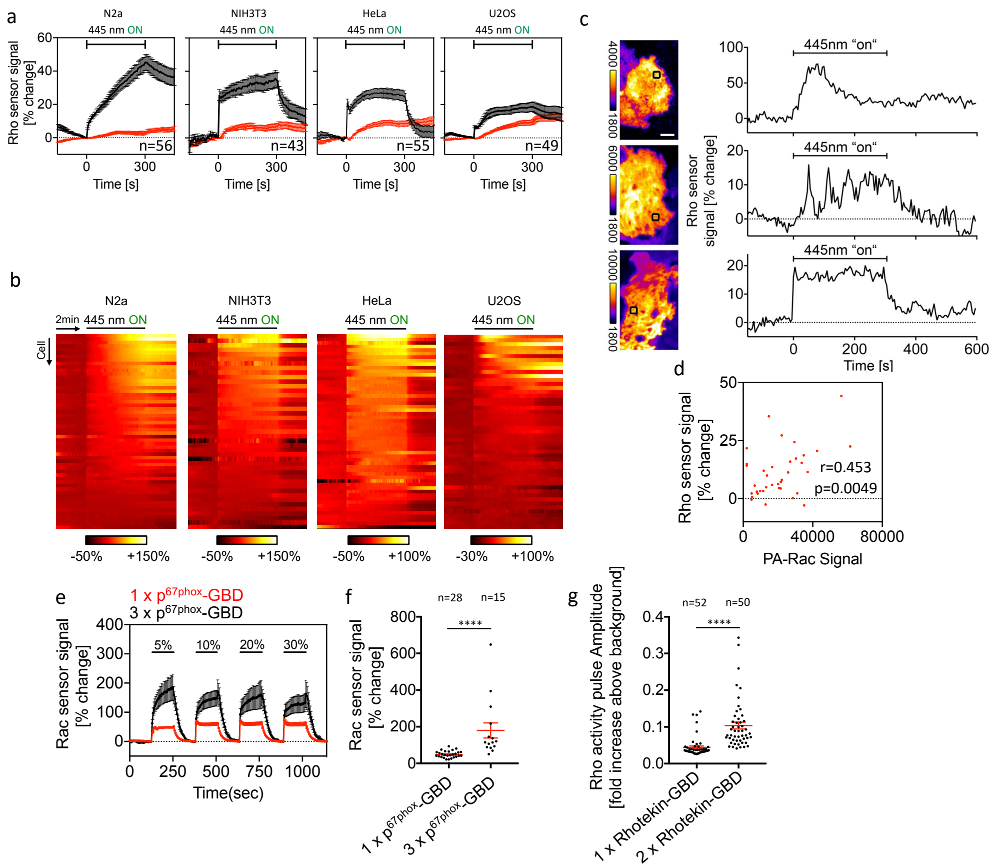

Supplementary Figure 3: Dynamic Rho activity response to rapid Rac1 activity perturbation. a: Measurement of average Rho activity sensor kinetics before, during and after Rac1 activation in N2a, NIH3T3, HeLa and U2OS cells. **b**: Heatmap representation of all Rho activity responses corresponding to a. Color represents % change of the Rho activity sensor. **c**: Examples of Rac1-induced Rho activity dynamics in individual U2OS cells (n=49 cells from 3 independent experiments). Numbers are indicated as percentage ± standard error of the mean. Left: Representative TIRF images. Right: Local Rho activity sensor measurements corresponding to black boxes in the TIRF images on the left. 56±11% of 44 cells showed a discernible Rho activity response. Top graph: transient single pulse response (46±30% of the 26 responding cells), Middle graph: increased pulse frequency/amplitude (24±12% of the 26 responding cells), Bottom graph: general Rho activity increase (30±20% of the 26 responding cells). All observations and measurements are based on at least 3 independent repetitions. **d**: Correlation of the Rho activity response with PA-Rac1 fluorescence signal intensity. Pearson's r and the corresponding p-value are indicated in the X-Y plot. **e-f**: Characterization of Rac activity sensors. **e**: Rac sensor response to rapid photoactivation via PA-Rac1 using increasing light intensities via neutral-density filters. **f**: Average sensor responses after 110s photoactivation with 5% light intensity. **g**: Measurement of Rho activity pulse amplitude in A431 cells that were stimulated by nocodazole application, according to previously published protocols and analyses[17]. Scale bar: 10 µm; 0.26µm/pixel. PA-Rac: Photoactivatable Rac; All statistical tests were two-sided. Source data are provided as a Source Data file.

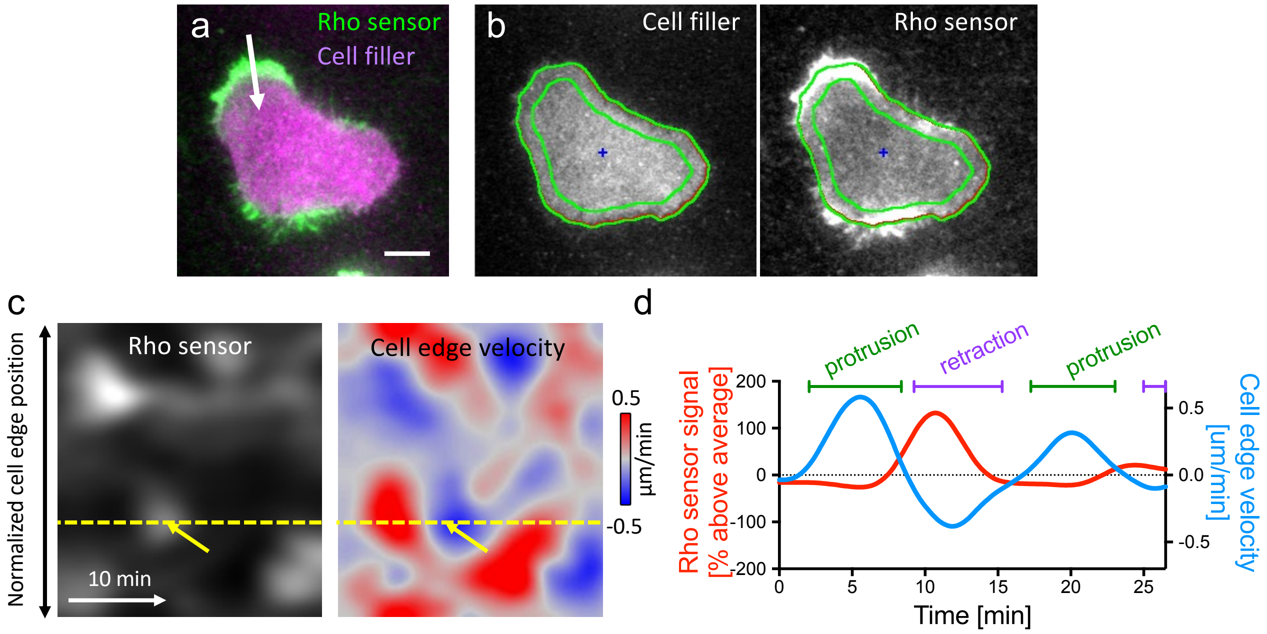

Supplementary Figure 4: Analysis of dynamic Rho enrichment in cell protrusion-retraction cycles. a: Representative A431 cell expressing a cytosolic cell volume marker (mCitrine) and an improved Rho activity sensor (mCherry-3xRhotekinGBD; top; n=21 cells from 3 independent experiments). The white arrow marks the direction of a local cell protrusion. **b**: Automated tracing of the cell border using a modified version of the ADAPT plugin [43]. **c**: Maps generated by the ADAPT plugin that represent the spatio-temporal dynamics of the Rho sensor signal between the green lines in **b** (left) and of the cell edge velocity (right). The yellow arrows in **c** point to the local protrusion that occurs at the position of the cell area marked by the yellow arrow in **b.** Red areas in the velocity map correspond to local cell protrusions, blue areas to local cell retractions. **d**: Plot of Rac sensor signals and cell edge velocity corresponding to the yellow dotted line in **c.** Values are normalized to average control sensor enrichment measurements. n=3 independent experiments with >21 cells per condition. Error bars represent standard error of the mean. Scale bars: 10µm; 0.26µm/pixel. Source data are provided as a Source Data file.

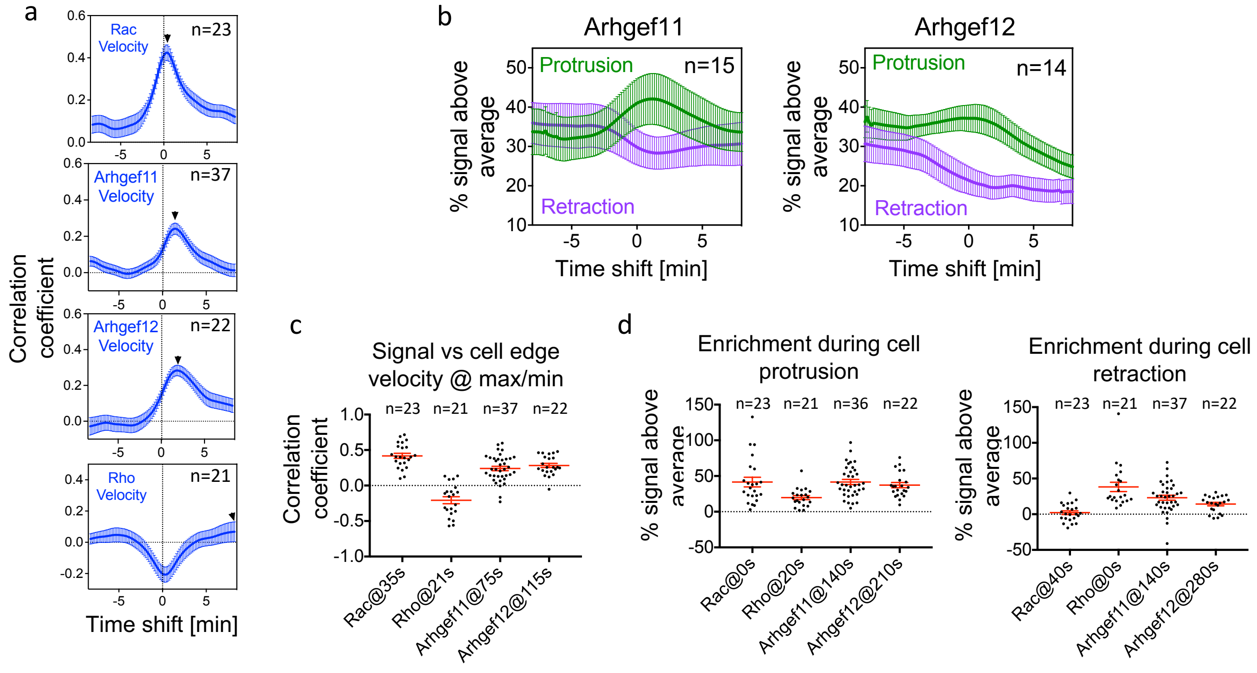

Supplementary Figure 5: Correlation of Arhgef11 and Arhgef12 signals with cell edge movements and enrichment in local cell protrusion-retraction cycles. a: Direct comparison of signal-cell edge velocity crosscorrelation functions for active Rac, Arhgef11, Arhgef12 and active Rho, related to Fig. 5e. Black arrows indicate the time point of maximal sensor or GEF correlation. The measurements shown in this panel are identical to measurements shown in Fig. 4f and Fig. 5d. **b**: Enrichment of Arhgef11/Arhgef12 signals in protrusions and retractions. Arhgef11/Arhgef12 enrichment values are normalized to average control construct enrichment measurements. The plasma membrane marker delCMV-mCitrine-CAAX ws used as control. n=3 independent experiments with >13 cells per condition. **c**: Quantification of the correlation coefficient at the respective maxima or minima of the correlation functions shown in a. **d**: Quantification of the signal enrichment during protrusion or retraction at the respective maxima or minima of the enrichment functions shown in Fig. 4g and Fig. 5d. (*: P<0.05; **: P<0.01; ***: P<0.001; One-way ANOVA). Error bars represent standard error of the mean. Source data are provided as a Source Data file.

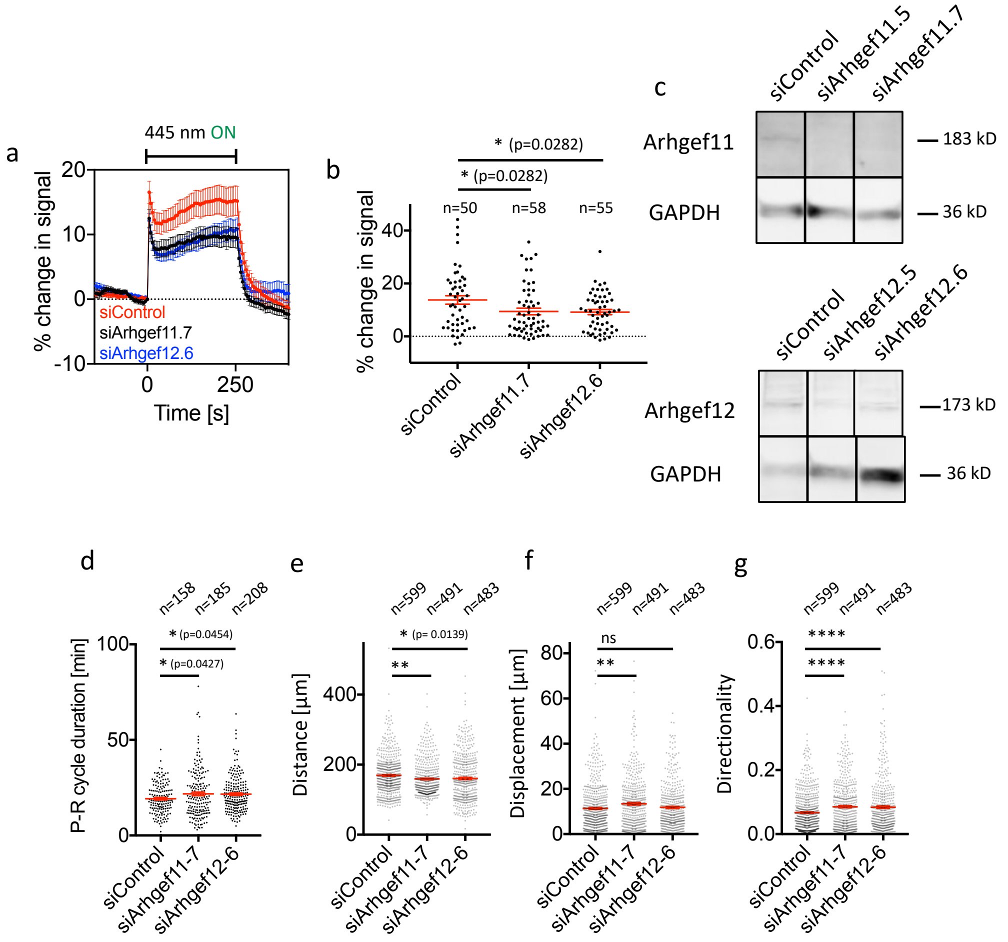

Supplementary Figure 6: Investigation of the role of Arhgef11 and Arhgef12 in Rac/Rho activity crosstalk, cell morphodynamics and cell migration. a,b: Quantification of average Rho activity sensor kinetics before, during and after Rac1 activation in A431 cells that co-express the Rho activity sensor, PA-Rac1 and control or Arhgef11/12 targeting siRNA oligonucleotides. **a**: Measurement of average Rho activity sensor kinetics. **b:** Quantification of the Rho activity response in the 25s time frame during photoactivation. **c**: Quantification of Arhgef11/12 knockdown via Western blot analysis. A representative blot is shown (n=3 independent repetitions). Quantification of knockdown efficiency: 83±15% for Arhgef11-5, 75±11% for Arhgef12-5, 80±10.3% for Arhgef11-7 and 70±16.7.% for Arhgef12-6 (percent ± standard error of the mean). **d**: Quantification of protrusion-retraction (P-R) cycle duration based on cell edge velocity measurements related to Fig. 6d using a second set of siRNA oligonucleotides (n=3 independent experiments with >158 cells per condition). **e-g**: Quantification of distance (e), displacement (f) and directionality (g), of A431 cell trajectories over a 4h time period in control and Arhgef11/Arhgef12 depleted cells, related to Fig. 6f-h, using a second set of siRNA oligonucleotides (n=3 independent experiments with >484 cells per condition). (*: P<0.05; **: P<0.01; ***: P<0.001; One-way ANOVA). Images were recorded at a frame rate of 1.5/min (d) or 1/min (e-g). Error bars represent standard error of the mean. All statistical tests were two-sided. Source data are provided as a Source Data file.
