# Supplementary material for: Rho GTPase activity crosstalk mediated by Arhgef11 and Arhgef12 coordinates cell protrusion-retraction cycles
Source: Nat Commun. 2023 Dec 15;14:8356. doi: 10.1038/s41467-023-43875-y (PMC10724141; doi:10.1038/s41467-023-43875-y)
Supplement: Supplementary file 3 — Description of Additional Supplementary Files [file 41467_2023_43875_MOESM3_ESM.pdf]

Description of Additional Supplementary Files

File Name: Supplementary Movie 1
Description: Morphological changes during rapid and reversible perturbation of the small GTPases Rac1, Cdc42 and RhoA via chemically-induced dimerization (related to Fig. 1b). Time-lapse TIRF videos of mCherry-Actin in representative Neuro-2a cells before and during the application of the chemical dimerizer SLF'-TMP and the competitor TMP, which induce reversible plasma membrane targeting of dominant positive mutants of the small GTPases Rac1, Cdc42 and RhoA. Images were collected with a frame rate of 2/min.

File Name: Supplementary Movie 2
Description: Direct investigation of Rac1-Rho crosstalk in living cells via chemically-induced dimerization (related to Fig. 2b). Time-lapse TIRF videos of the perturbation construct mTurquoise2-Rac1Q61LΔCAAX, the Rho activity sensor mCherry-Rhotekin-GBD and the control sensor mCitrine in a representative Neuro-2a cell before and during the application of the chemical dimerizer SLF'-TMP and the competitor TMP. Images were collected with a frame rate of 3/min.

File Name: Supplementary Movies 3 and 4
Description: Rac activity dynamics in protrusion-retraction cycles in spontaneously migrating A431 cells (related to Fig. 4a). Time-lapse TIRF videos of the improved Rac activity sensor (mCherry-3xp67^{phox}GBD; green) in A431 cells. A cytosolic cell volume marker (mCitrine) was co-expressed to detect the cell attachment area (magenta). Images were collected with a frame rate of 6/min.

File Name: Supplementary Movie 5
Description: Rho activity dynamics in protrusion-retraction cycles in spontaneously migrating A431 cells (related to Fig. 4e). Time-lapse TIRF videos of the improved Rho activity sensor (mCherry-2X-RhotekinGBD; green) in a A431 cell. A cytosolic cell volume marker (mCitrine) was co-expressed to detect the cell attachment area (magenta). Images were collected with a frame rate of 6/min.

File Name: Supplementary Movie 6
Description: Arhgef11 plasma membrane association dynamics in protrusion-retraction cycles in spontaneously migrating A431 cells (related to Fig. 5c). Time-lapse TIRF videos of a fluorescently labeled Arhgef11 construct (mCherry-Arhgef11; green) in a A431 cell. A cytosolic cell volume marker (mCitrine) was co-expressed to detect the cell attachment area (magenta). Images were collected with a frame rate of 6/min.

File Name: Supplementary Movie 7

Description: Arhgef12 plasma membrane association dynamics in protrusion-retraction cycles in spontaneously migrating A431 cells (related to Fig. 5c). Time-lapse TIRF videos of a fluorescently labeled Arhgef12 construct (mCherry-Arhgef12; green) in a A431 cell. A cytosolic cell volume marker (mCitrine) was co-expressed to detect the cell attachment area (magenta). Images were collected with a frame rate of 6/min.
